# Efficacy of Cerebral Embolic Protection Device in Transcatheter Aortic Valve Replacement: A Systematic Review and Meta‐Analysis

**DOI:** 10.1002/ccd.70146

**Published:** 2025-09-05

**Authors:** Sufyan Shahid, Mahrosh Kasbati, Samer Osama Adawi, Saniya Ishtiaq, Muhammad Osama, Syed Mohamin Abbas Shah, Muneeb Saifullah, Syed Ali, Raheel Ahmed, Iftikhar Ali Ch, Salman Khalid, Naeem Khan Tahirkheli

**Affiliations:** ^1^ Khawaja Muhammad Safdar Medical College Sialkot Pakistan; ^2^ Dow University of Health Sciences Karachi Pakistan; ^3^ Al‐Quds University Jerusalem Palestine; ^4^ Rawalpindi Medical University Rawalpindi Pakistan; ^5^ Hayatabad Medical Complex Peshawar Pakistan; ^6^ King Edward Medical University Lahore Pakistan; ^7^ Oklahoma Heart Hospital Oklahoma City Oklahoma USA; ^8^ National Heart and Lung Institute Imperial College London London UK; ^9^ South Oklahoma Heart Research Oklahoma City Oklahoma USA

**Keywords:** CEPD, cerebral embolic protection device, embolic events, meta‐analysis, neurological complications, stroke prevention, systematic review, TAVR, transcatheter aortic valve replacement

## Abstract

Transcatheter aortic valve replacement (TAVR) is widely used to treat severe aortic stenosis; however, periprocedural stroke remains a significant concern. This systematic review and meta‐analysis evaluate whether the use of cerebral embolic protection devices (CEPDs) during TAVR reduces the risk of stroke and other complications. To conduct a network meta‐analysis of relevant trials to assess the efficacy of CEPDs currently used in TAVR. PubMed, Embase, and Scopus were systematically searched through April 2025 to identify studies comparing TAVR performed with and without CEPDs. Primary outcomes included stroke, all‐cause mortality, major bleeding, and major vascular complications. Data were analyzed using RevMan (Version 5.4.1). A random‐effects model was used for all analyses, applying the Mantel−Haenszel method for dichotomous outcomes, reported as risk ratios with 95% confidence intervals. Funnel plots were used to assess publication bias. Twenty‐four studies (9 randomized controlled trials and 15 observational studies), including a total of 437,487 patients (59,274 with CEPD and 384,213 without), were included in the analysis. The mean patient age was 80 years, and 46.4% were female. Compared to patients undergoing TAVR without protection, those receiving CEPDs had significantly lower risks of stroke (RR = 0.70; 95% CI: 0.60–0.82; *p* < 0.0001), all‐cause mortality (RR = 0.69; 95% CI: 0.50–0.93; *p *= 0.02), disabling stroke (RR = 0.44; 95% CI: 0.26–0.75; *p* = 0.003), acute kidney injury (RR = 0.84; 95% CI: 0.79–0.89; *p* < 0.00001), and 30‐day readmission (RR = 0.75; 95% CI: 0.60–0.95; *p* = 0.02). A reduction in major bleeding was also observed (RR = 0.83; 95% CI: 0.59–1.17), although this did not reach statistical significance (*p* = 0.29). No significant differences were found between groups in terms of major vascular complications, transient ischemic attack (TIA), new pacemaker implantation, or delirium. The use of CEPDs during TAVR is associated with reduced risks of stroke, disabling stroke, acute kidney injury, and 30‐day readmission. However, discrepancies between randomized and observational studies warrant cautious interpretation. Further research is needed to clarify the benefits and evaluate the cost‐effectiveness of CEPD implementation in routine clinical practice.

**Abbreviations**
AKI: acute kidney injury
CEPDs: cerebral embolic protection devices
CI: confidence interval
PPM: permanent pacemaker
RCTs: randomized controlled trials
RevMan: Review Manager
RR: risk ratio
SD: standard deviation
TAVR: transcatheter aortic valve replacement
TIA: transient ischemic attack

## 1 Introduction

Transcatheter aortic valve replacement (TAVR) is a well‐established intervention for severe, symptomatic aortic stenosis, which is the most common valvular disease in Europe and North America [1]. It entails the insertion of a prosthetic valve via a catheter, without removing the diseased valve. The implanted valve is usually inserted through the femoral artery, although there are alternative vascular access options such as transapical and transaortic approaches as well [2]. In patients at high or prohibitive surgical risk, TAVR has shown superiority over both medical therapy and surgical aortic valve replacement [3], and it has been associated with lower 1‐year mortality, improved cardiac symptoms, and enhanced hemodynamic performance [4]. However, TAVR is not without complications, which may include paravalvular leak, stent migration, and stroke [1].

Post‐TAVR stroke, although relatively infrequent, is associated with increased morbidity and mortality [5], making stroke prevention a clinical priority. For this purpose, cerebral embolic protection devices (CEPDs) have been developed. They have been designed to capture or deflect embolic debris released during TAVR [6]. Although several CEPDs such as the TriGUARD 3 and EMBOL‐X have been developed and are being studied, only the Sentinel CEP device (Boston Scientific) has been approved for clinical use in both the United States and Europe [7].

Although CEPDs have been proposed as a means to reduce ischemic brain injury during transcatheter aortic valve implantation (TAVI), the evidence supporting their clinical benefit remains inconclusive. While some trials, such as SENTINEL, suggest a potential reduction in stroke risk, systematic reviews and meta‐analyses to date have not demonstrated a consistent or statistically significant benefit [8, 9]. Moreover, uncertainties persist regarding their cost‐effectiveness, real‐world clinical utility, and the identification of patients who are most likely to benefit. This inconsistency in the evidence base reveals a critical gap in our understanding of the true value of CEPDs in TAVI. To address this, our systematic review and meta‐analysis integrate data from both randomized controlled trials (RCTs) and observational studies, aiming to provide a more robust and comprehensive evaluation of CEPD efficacy by increasing statistical power and improving generalizability.

## 2 Methods

This systematic review and meta‐analysis was conducted in accordance with the PRISMA guidelines to ensure transparency and high‐quality reporting (1). The study protocol has been officially registered with the International Prospective Register of Systematic Reviews (PROSPERO) under the ID: CRD420251039917.

### 2.1 Search Strategy

Several databases including PubMed, Embase, and Scopus were systematically searched without any language restrictions covering publications up to April 11, 2025. The MeSH terms and keywords that were used included: “Transcatheter Aortic Valve Replacement,” “TAVR,” “Cerebral Embolic Protection Devices,” “Embolic Protection Devices,” “Cerebral Protection Device,” and “CEPDs.” Relevant articles were also looked through in the bibliographies of all the included papers. The detailed search strategy for individual databases is provided in Supporting Information S1: Table 1.

### 2.2 Study Selection and Eligibility Criteria

All references identified during our search were first imported into EndNote v20, where we removed any duplicates. Then, two authors (S.A. and M.O.) independently screened the titles and abstracts to exclude studies that didn't meet our inclusion criteria. For the studies that passed this stage, we carefully reviewed the full texts to determine their eligibility. Any disagreements were resolved through discussion with a senior author (S.S.). We included both observational studies and RCTs that compared outcomes between patients who underwent TAVR with CEPD and those who did not.

### 2.3 Data Extraction and Outcomes

Two authors (S.A. and M.O.) extracted the data into Microsoft Excel, and in cases of discrepancies, a third author (S.S.) was consulted. The data includes the trial name, first author, publication year, study setting, number of patients in the study, and each treatment group. Patient characteristics included age, sex, history of previous MI, previous CABG, transient ischemic attack (TIA), and stroke. The outcomes assessed in this meta‐analysis included stroke, disabling stroke, all‐cause mortality, major vascular complications, acute kidney injury (AKI), major bleeding, TIA, new permanent pacemaker (PPM) placement, delirium, and 30‐day readmission. All the included RCTs adhered to the Valve Academic Research Consortium updated definitions (VARC) for reporting clinical outcomes.

### 2.4 Quality Assessment

Two independent reviewers (M.K. and M.O.) evaluated the risk of bias using RoB 2.0 for RCTs and ROBINS‐I for observational studies. For RCTs, the following domains were assessed in accordance with the RoB 2.0 tool: (1) bias arising from the randomization process, (2) bias due to deviations from intended interventions, (3) bias due to missing outcome data, (4) bias in measurement of the outcome, and (5) bias in selection of the reported result. For observational studies, the ROBINS‐I tool was applied to evaluate bias across seven domains: (1) confounding, (2) selection of participants, (3) classification of interventions, (4) deviations from intended interventions, (5) missing data, (6) measurement of outcomes, and (7) selection of the reported result. Based on these criteria, each study was rated as having a low risk of bias, some concerns, or a high risk of bias. Any disagreements were resolved through discussion with a third reviewer (S.S.). Funnel plots were used to evaluate potential publication bias.

### 2.5 Statistical Analysis

We carried out the statistical analysis using Review Manager (RevMan) version 5.4.1. For continuous outcomes, we applied the inverse variance method and reported results as mean ± standard deviation (SD) along with 95% confidence intervals (CI). For dichotomous outcomes, we used the Mantel−Haenszel method and expressed the findings as risk ratios (RR) with 95% CI. A random‐effects model was chosen to account for differences among the studies. The combined results were visually presented using forest plots. *I*² statistics were used to assess how much the study results varied from each other. We also performed subgroup analyses based on the type of study design (RCTs vs. observational studies).

## 3 Results

### 3.1 Search Results

**Figure 1:**
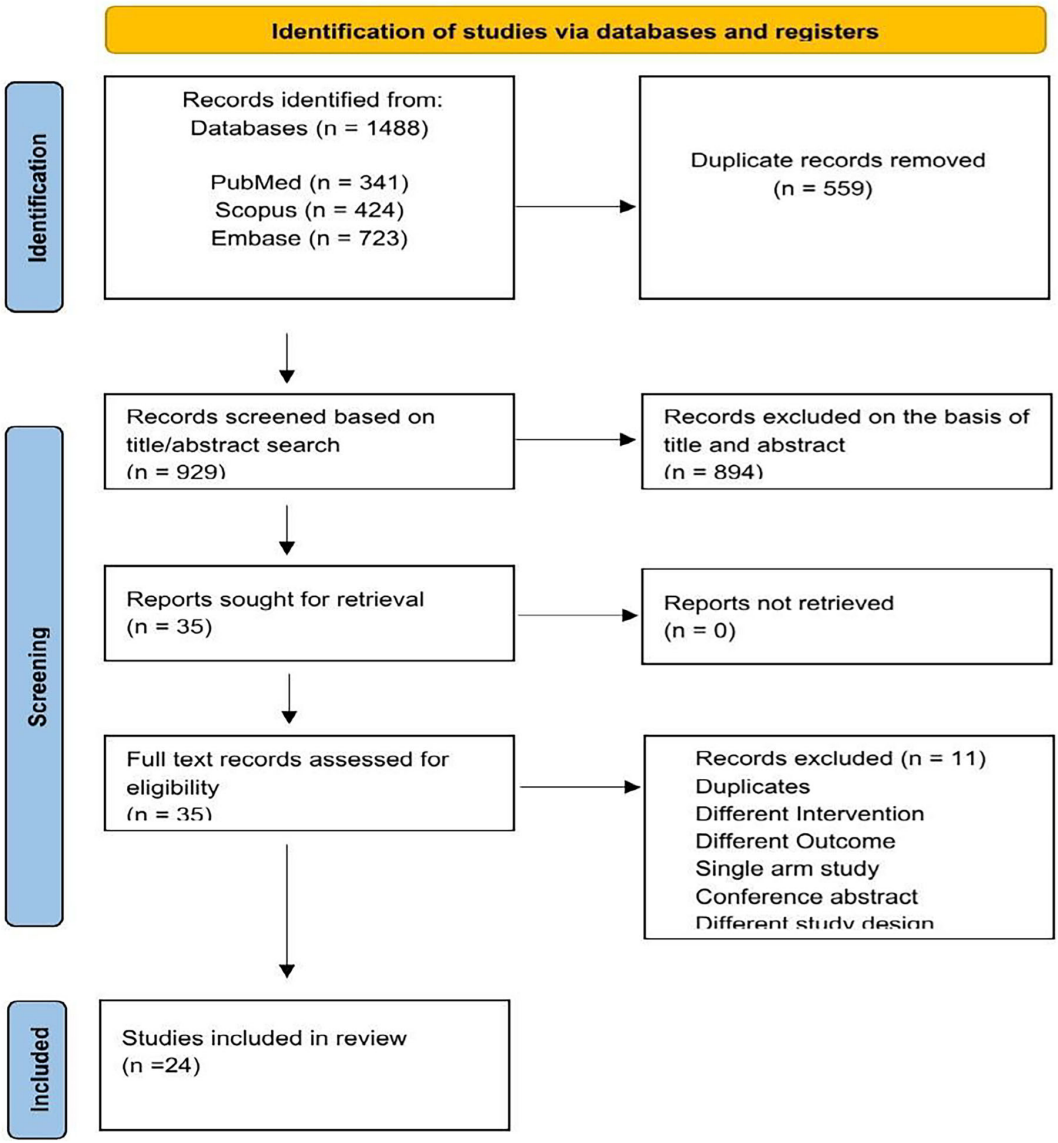
PRISMA flowchart of the screening process. [Color figure can be viewed at wileyonlinelibrary.com]

The preliminary search yielded 1488 articles. After filtering out duplicates, we evaluated the remaining 929 articles to determine their eligibility. A total of 894 publications were excluded after reviewing their titles and abstracts. The remaining 35 articles were assessed for eligibility criteria. After full‐text assessment, a total of 24 studies were included in the analysis. Figure 1 provides an in‐depth summary of the screening process.

### 3.2 Study Characteristics

Out of 24 studies, 9 were RCTs and 15 were observational studies. The publication years spanned from 2016 to 2025. These studies covered a total of 437,487 patients, with 59,274 receiving CEPD and 384,513 receiving non‐CEPD. The mean age of the patients is 80.08 years. Out of these, 3058 (46.42%) were of the female gender. The countries of origin included the USA (9), Europe (2), Germany (6), the Netherlands (2) UK (2), China (1), Austria (1), and one had patients from multiple countries. The Sentinel device was the most commonly used, reported in 11 studies. Other devices included TriGuard (HDH or 3) in four studies, Montage in one study, EMBOL‐X in one study, while five studies did not report the device used. The detailed baseline characteristics of included studies are provided in Table 1.

**Table 1:** Baseline characteristics of studies comparing cerebral embolic protection devices (CEPD) versus no CEPD.

Study	Country	Study type	Total patients	CEPD (*n*)	No CEPD (*n*)	Follow‐up duration	Device used	Mean age (years)		Female (%)		Prior MI (%)		Prior PCI (%)		Prior CABG (%)		Prior stroke (%)		Heart failure (%)		Renal disease (%)	
								CEPD	No CEPD	CEPD	No CEPD	CEPD	No CEPD	CEPD	No CEPD	CEPD	No CEPD	CEPD	No CEPD	CEPD	No CEPD	CEPD	No CEPD
*Randomized clinical trials*																							
PROTECTED‐TAVR 2022	International	RCT	3000	1501	1499	72 h or before discharge	Sentinel	78.9	78.9	37.8	42.0	NR	NR	NR	NR	NR	NR	7.6	8.2	NR	NR	NR	NR
REFLECT I 2021	US/Europe	RCT	204	141	63	In‐hospital, at 30 days and at 90 days	TriGuard HDH	79.8	81.5	43.3	33.3	26.2	30.0	21.5	16.1	7.5	6.7	7.7	6.7	17.1	10.2	19.9	17.7
REFLECT II 2021	USA	RCT	214	157	57	Both In‐hospital and at 30 days	TriGuard 3	80.3	78.1	45.2	38.6	31.2	26.3	18.5	19.3	10.8	3.5	7.8	3.5	19.9	23.2	22.9	29.8
CLEAN‐TAVI 2016	Germany	RCT	100	50	50	2, 7, and 30 days for stroke, 30 days (others)	Montage	80.0	79.3	58.0	56.0	12.0	8.0	10.0	16.0	16.0	4.0	NR	NR	92.0	92.0	86.0	78.0
MISTRAL‐C 2016	Netherlands	RCT	65	32	33	30 days (for mortality, 5 days, 30 days, and 6 months)	Sentinel	82.0	82.0	47.0	49.0	6.0	6.0	NR	NR	NR	NR	NR	NR	NR	NR	NR	NR
DEFLECT III 2015	Europe	RCT	85	46	39	In‐hospital and at 30 days	TriGuard HDH	82.5	82.3	56.5	51.3	13.0	21.1	30.4	46.2	10.9	7.7	NR	NR	45.4	38.5	23.9	25.6
BHF PROTECT‐TAVI2025	UK	RCT	7635	3798	3803	72 h or until discharge, for disabling stroke, also 6−8 weeks	Sentinel	81.2	81.2	39.1	38.4	NR	NR	NR	NR	NR	NR	6.3	6.3	14.1	12.8	NR	NR
SENTENIL 2017	USA/Germany	RCT	240	121	119	30 days	Sentinel	81.6	82.5	51.0	45.0	17.4	16.8	18.2	21.0	4.1	5.0	7.4	6.8	NR	NR	NR	NR
EMBOL‐X 2015	USA	RCT	30	14	16	7 days	EMBOL‐X	81	82.1	71.4	50	NR	NR	NR	NR	NR	NR	7.1%	12.5	NR	NR	42.8	37.5
*Observational studies*																							
Zhang 2023	China	Retrospective PSM	2385	795	1590	In‐hospital	NR	70.5	69.4	37.7	38.7	8.2	7.2	16.4	13.2	3.8	3.8	9.4	11.6	74.2	73.0	17.0	21.4
Isogai 2022	USA	Retrospective	2839	1802	1037	72 h or until discharge		79.0	79.4	40.0	44.0	22.2	22.1	29.7	29.0	28.1	28.7	12.6	12.3	78.2	78.9	3.6	3.1
Lind 2022	Germany	Retrospective	51	18	33	NR	TriGuard 3	91.7	NR	55.6	57.6	38.9	57.5	NR	NR	44.4	15.1	NR	NR	55.6	66.6	NR	NR
Yashima 2022	USA	Retrospective PSM	7610	3805	3805	In‐hospital or until discharge		79.3	78.9	44.5	41.9	13.0	13.3	NR	NR	12.6	15.1	14.6	15.6	74.8	78.7	33.1	35.6
Dona 2022	Austria	Prospective	411	213	198	72 h	Sentinel	80.4	80.4	44.6	50.5	NR	NR	NR	NR	NR	NR	7.1	7.7	60.3	66.1	15.2	11.7
Voss 2019	Germany	Retrospective	391	39	352	Intra‐operative	Sentinel	79.1	79.5	53.8	50.5	NR	NR	NR	NR	NR	NR	5.1	8.0	NR	NR	NR	NR
Stamou 2019	USA	Retrospective	83	33	50	NR	NR	NR	NR	NR	NR	9.0	24.0	NR	NR	NR	NR	NR	NR	NR	NR	NR	NR
Seeger 2018	Germany	Retrospective PSM	1066	533	533	72 h	Sentinel	81.2	81.0	52.9	52.9	23.3	25.3	12.6	13.1	NR	NR	NR	NR	NR	NR	NR	NR
Seeger 2017	Germany	Prospective	560	280	280	7 days	Sentinel	80.6	80.9	54.3	55.0	NR	NR	8.6	12.9	9.3	13.6	NR	NR	7.1	11.8	28.9	29.6
Altibi 2024	USA	Retrospective	21,044	10,503	10,541	In‐hospital	Sentinel	78.9	78.6	40.7	40.6	22.4	21.8	13.4	13.5	15.5	14.5	NR	NR	90.0	89.7	NR	NR
Shekhar 2024	USA	Retrospective	100,928	6916	94,012	In‐hospital	NR	80.4	80.4	41.5	41.8	21.9	21.7	15.6	14.9	0.6	0.4	NR	NR	2.9	2.8	NR	NR
Shashank Shekhar 2024	USA	Retrospective	271,804	19,821	251,983	In‐hospital	Sentinel	78.8	78.8	44.2	44.6	21.5	21.6	16.2	14.0	0.5	0.5	NR	NR	2.3	3.7	5.8	6.5
Kroon 2019	Netherlands	PSM registry	666	333	333	30 days	Sentinel	81.0	81.0	50.0	49.0	45.0	44.0	NR	NR	NR	NR	10.0	11.0	NR	NR	NR	NR
Kemp 2021	NR	Retrospective	157	78	79	NR		NR	NR	NR	NR	NR	NR	NR	NR	NR	NR	NR	NR	NR	NR	NR	NR
Khan 2021	USA	Retrospective	108,315	4380	10,395	NR	NR	81.0	81.0	45.0	46.4	NR	NR	NR	NR	NR	NR	14.7	14.0	74.3	74.4	3.2	32.2

Abbreviations: CABG = coronary artery bypass grafting, CEPD = cerebral embolic protection device, MI = myocardial infarction, NR = not reported, PCI = percutaneous coronary intervention, PSM = propensity score matched, RCT = randomized clinical trial.

### 3.3 Quality and Publication Bias Assessment

The risk of bias was assessed using the RoB 2 tool for RCTs and the ROBINS‐I tool for observational studies. Among the RCTs, two studies (Kapadia 2022 and Lansky 2021) had low risk of bias across all domains, while the remaining showed some concerns, mostly related to the randomization process and deviations from intended interventions. For observational studies, most had low risk in outcome measurement and data completeness, but moderate risk in confounding and participant selection. Three studies (Lind 2022, Voss 2019, and Kemp 2022) were judged to have serious overall risk due to uncontrolled confounding. Only two observational studies were rated as low risk overall, with the rest showing moderate risk reflecting common limitations in non‐randomized designs (Supporting Information S1: Figures S1−S4). Publication bias was evaluated using funnel plots. The plots appeared symmetrical, with a substantial number of studies clustered near the center, indicating an even distribution of effect sizes and a minimal risk of publication bias in the included studies (Supporting Information S1: Figures S12−S22).

### 3.4 Primary Outcomes

1. Stroke

The outcome of stroke is reported by 23 studies (9 RCTs and 14 observational studies), including 360,571 patients (55,477 CEPD vs. non‐CEPD 305,094). Stroke incidence was significantly lower in patients who received CEPDs than in those who did not (RR = 0.70; 95% CI: 0.60–0.82; *p* < 0.0001; *I*² = 51%).

2. All‐cause mortality

This outcome is reported by 20 studies (8 RCTs and 12 observational studies), including 360,305 patients (55,355 CEPD vs. non‐CEPD 304,950). The analysis demonstrated that all‐cause mortality was significantly lower in the CEPD group compared to the non‐CEPD group (RR = 0.69; 95% CI: 0.50–0.93; *p* =  0.02; *I*² = 71%).

3. Major bleeding

This outcome is reported by 11 studies (5 RCTs and 6 observational studies), including 117,701 patients (9001 CEPD vs. non‐CEPD 108,700). Major bleeding rates were comparable between the CEPD and non‐CEPD groups, with no statistically significant difference observed (RR = 0.83; 95% CI: 0.59–1.17; *p* =  0.29; *I*² = 42%).

4. Major vascular complications

This outcome is reported by 16 studies (8 RCTs and 8 observational studies), including 54,279 patients (22,324 CEPD vs. non‐CEPD 31,955). No significant difference was found in the incidence of major vascular complications between the CEPD and non‐CEPD groups (RR = 0.90; 95% CI: 0.73–1.11; *p* =  0.32; *I*² = 43%).

### 3.5 Secondary Outcomes

1. Disabling stroke
  This outcome is reported by 14 studies (9 RCTs and 5 observational studies), including 14,386 patients (7344 CEPD vs. non‐CEPD 7042). The analysis demonstrated that the incidence of disabling stroke was significantly lower in the CEPD group compared to the non‐CEPD group (RR = 0.44; 95% CI: 0.26–0.75; *p*= 0.003; *I*² = 34%, Supporting Information S1: Figure S5).
2. Non‐disabling stroke
  This outcome is reported by 13 studies (8 RCTs and 5 observational studies), including 6893 patients (3549 CEPD vs. non‐CEPD 3344). There was no significant difference in the incidence of non‐disabling stroke between the CEPD and non‐CEPD groups (RR = 0.83; 95% CI: 0.60–1.14; *p* = 0.24; *I*² = 0%; Supporting Information S1: Figure S6).
3. TIA
  This outcome is reported by 8 studies (4 RCTs and 4 observational studies), including 125,353 patients (12,978 CEPD vs. non‐CEPD 112,375). TIA incidence was comparable between patients with and without CEPDs, with no statistically significant difference observed (RR = 0.88; 95% CI: 0.59–1.32; *p* = 0.54; *I*² = 0%; Supporting Information S1: Figure S7).
4. AKI
  This outcome is reported by 13 studies (7 RCTs and 6 observational studies), including 51,461 patients (21,477 CEPD vs. non‐CEPD 29,984). The analysis showed that the incidence of AKI in the CEPD group was significantly lower compared to non‐CPED groups (RR 0.84, 95% CI: 0.76−0.92, *p* = 0.0003, *I* ^2^ = 20%, Supporting Information S1: Figure S8).
5. Delirium
  This outcome is reported by 3 studies (2 RCTs and 1 observational study), including 3116 patients (1551 CEPD vs. non‐CEPD 1565). Both groups showed similar rates of delirium, with no significant association between CEPD use and this outcome (RR = 0.71; 95% CI: 0.31–1.63; *p* = 0.42; *I*² = 20%; Supporting Information S1: Figure S9).
6. 30‐day readmission
  This outcome is reported by 3 studies, including 123,230 patients (23,830 CEPD vs. non‐CEPD 99,400). Patients receiving CEPDs had a significantly reduced risk of 30‐day readmission compared to those without CEPDs (RR = 0.75; 95% CI: 0.60–0.95; *p* = 0.02; *I*² = 93%; Supporting Information S1: Figure S10).
7. New PPM placement
  This outcome is reported by 7 studies (5 RCTs and 2 observational studies), including 22,062 patients (10,855 CEPD vs. non‐CEPD 11207). New PPM implantation rates were comparable between patients with and without CEPDs, with no statistically significant difference observed (RR = 1.07; 95% CI: 0.99–1.16; *p* = 0.09; *I*² = 0%; Supporting Information S1: Figure S11).

### 3.6 Subgroup Analysis

**Figure 2:**
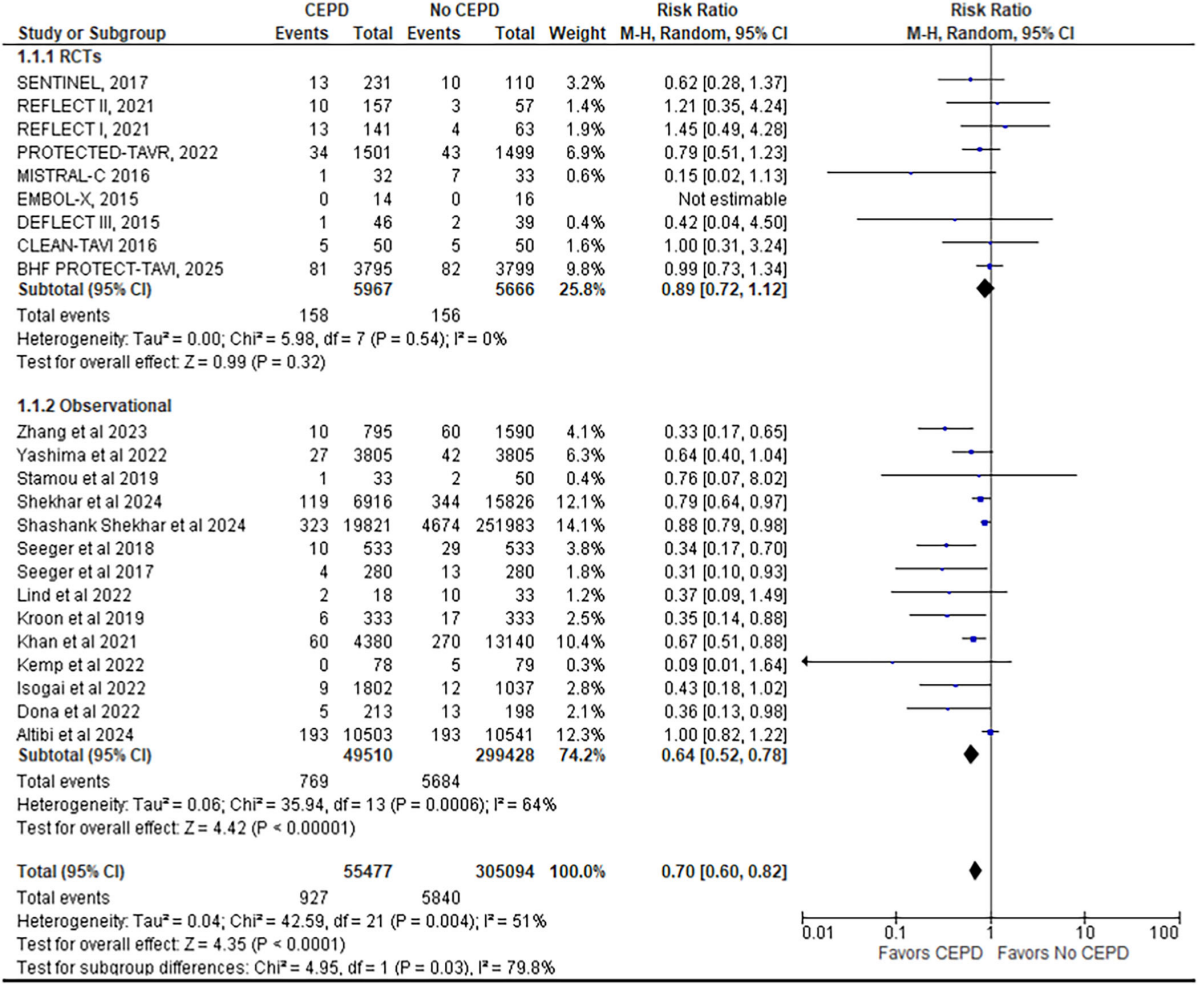
Forest plot comparing the outcome of stroke for patients who underwent TAVR with cerebral embolic protection device (CEPD) to those who underwent TAVR without the use of CEPD. [Color figure can be viewed at wileyonlinelibrary.com]

**Figure 3:**
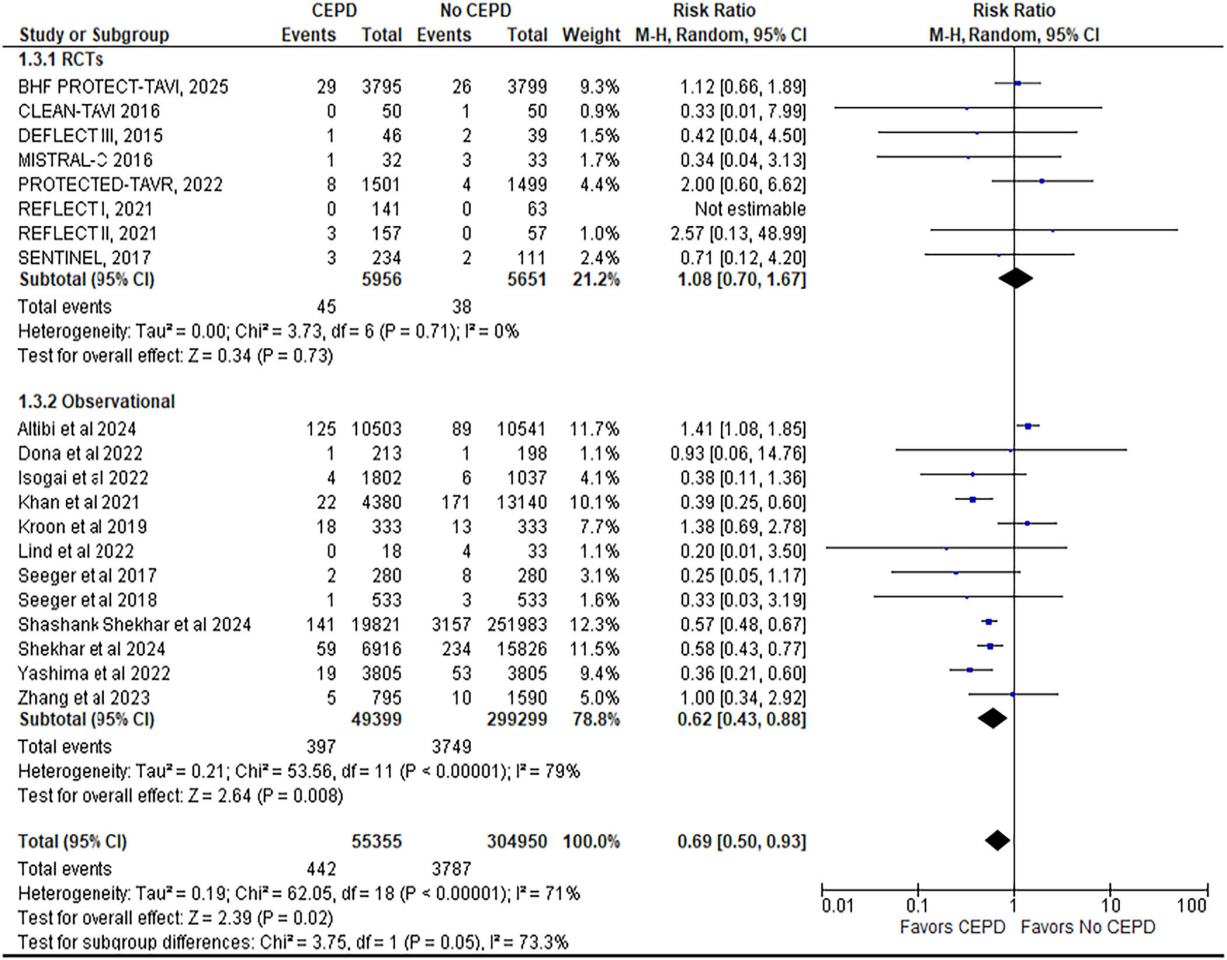
Forest plot comparing the outcome of all‐cause mortality for patients who underwent TAVR with cerebral embolic protection device (CEPD) to those who underwent TAVR without the use of CEPD. [Color figure can be viewed at wileyonlinelibrary.com]

**Figure 4:**
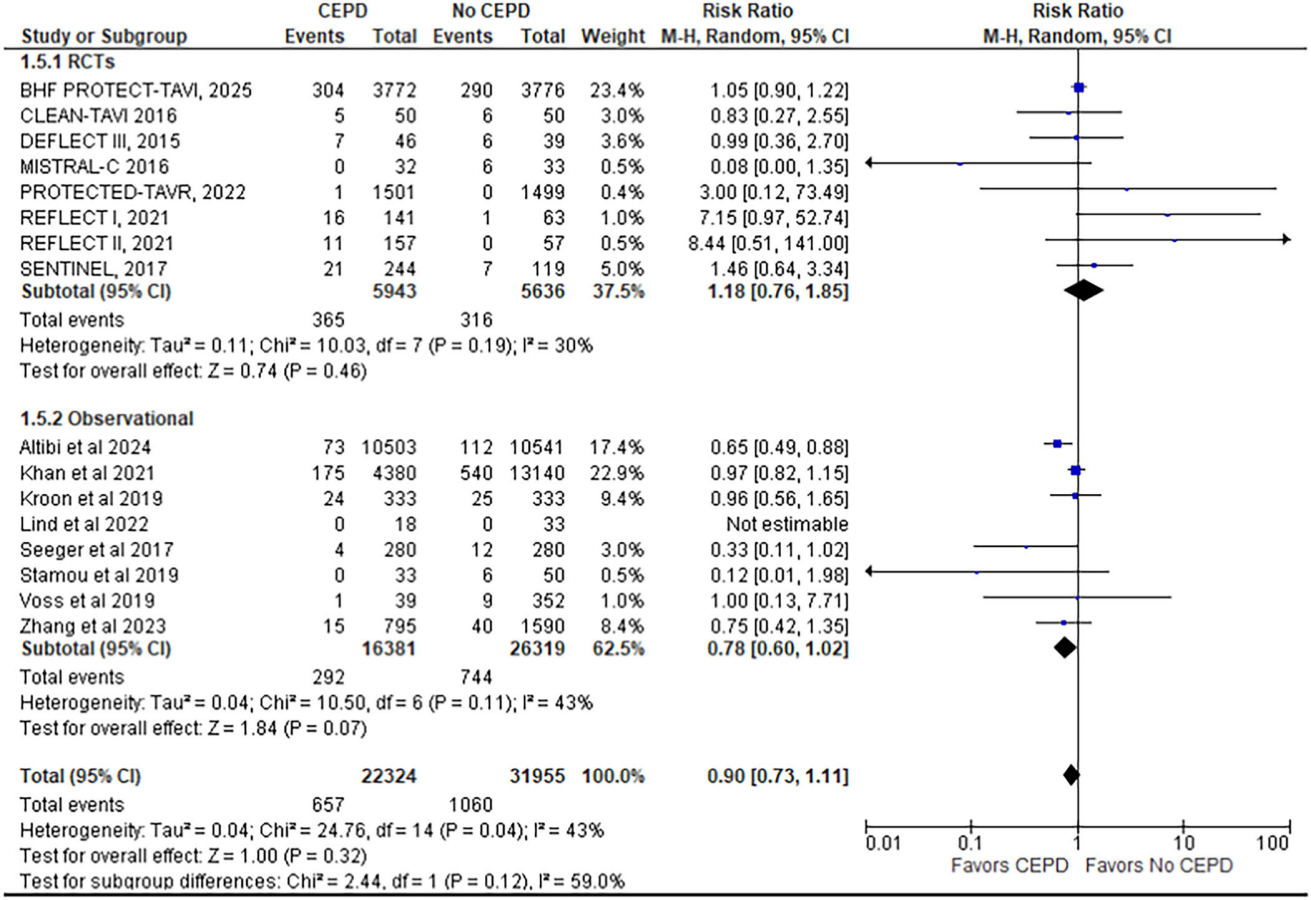
Forest plot comparing the outcome of major vascular complications for patients who underwent TAVR with cerebral embolic protection device (CEPD) to those who underwent TAVR without the use of CEPD. [Color figure can be viewed at wileyonlinelibrary.com]

A subgroup analysis was conducted to evaluate the efficacy of CEPDs based on study design, specifically comparing RCTs with observational studies. Of the 24 studies included in the meta‐analysis, 8 were RCTs and 16 were observational studies. The subgroup analysis demonstrated that the benefit of CEPD use in reducing stroke, disabling stroke, all‐cause mortality, and major vascular complications was observed only in observational studies and not in RCTs, with a statistically significant difference between the two study designs (*p* < 0.05) (Figures 2, 3, 4 and Supporting Information S1: Figure S5).

**Figure 5:**
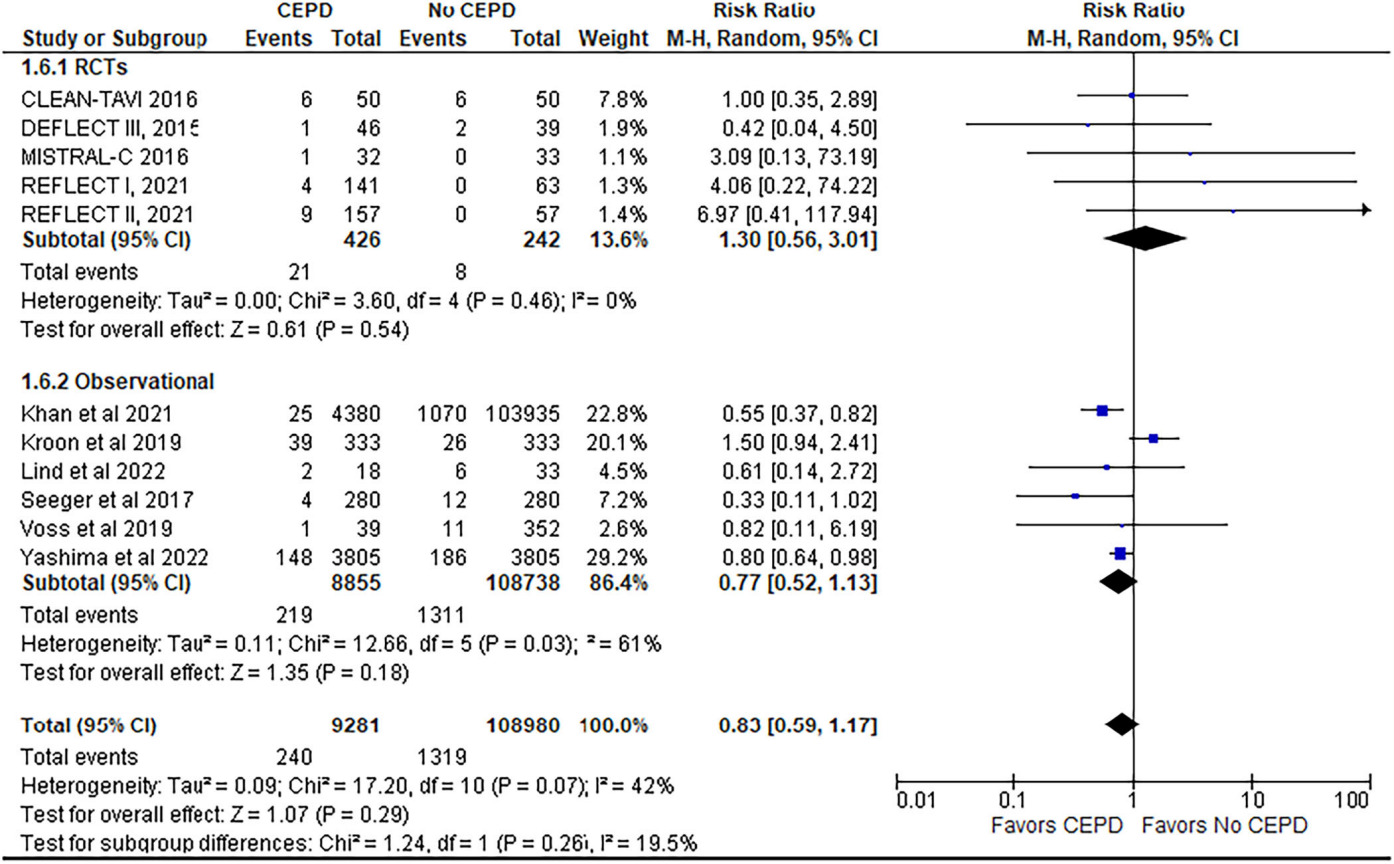
Forest plot comparing the outcome of major bleeding for patients who underwent TAVR with a cerebral embolic protection device (CEPD) to those who underwent TAVR without the use of CEPD. [Color figure can be viewed at wileyonlinelibrary.com]

For other outcomes such as major bleeding, TIA, and AKI, a reduction was also seen only in observational studies; however, the difference between observational studies and RCTs was not statistically significant (*p* > 0.05) (Figure 5, Supporting Information S1: S6−S8). Outcomes such as delirium and PPM implantation were comparable between CEPD and non‐CEPD groups across both RCTs and observational studies, showing no significant differences in either study design (Supporting Information S1: Figures S9, S11).

## 4 Discussion

This systematic review and meta‐analysis, encompassing a total of 437,457 patients across 24 studies, examined all currently available data on CEPD use in patients undergoing TAVR. Our findings indicated that CEPD use is associated with statistically significant reductions in the incidence of stroke, disabling stroke, AKI, major bleeding, and 30‐day hospital readmission. These outcomes demonstrate the potential protective role of CEPDs in not just preventing neurologic complications but also in reducing systemic embolic events and morbidity. However, it is important to note that the rates of vascular complications, TIA, new PPM implantation, and postoperative delirium were comparable between groups with and without CEPD use.

Stroke is by far the most serious complication of TAVI with a 30‐day incidence of approximately 2.3%. Early meta‐analyses assessing CEPDs did not demonstrate a significant reduction in clinical stroke rates [8, 10, 11, 12]. However, other studies suggest that CEPDs may offer benefits in reducing stroke and the volume of cerebral ischemic lesions. For instance, the CLEAN‐TAVI and MISTRAL‐C trials [13, 14] reported that the use of a CEPD reduced both the number and volume of new cerebral lesions detected by MRI in patients undergoing TAVR. The pathophysiology of stroke in TAVR patients is multifactorial, involving both patient‐related and procedural factors. Patient‐related risk factors include advanced age, prior cerebrovascular events, atrial fibrillation, and chronic kidney disease. Procedural factors include manipulation of calcified aortic valves, which can dislodge debris [15, 16], and the presence of complex aortic arch atheroma [17], which increases the chances of embolization during catheter navigation. Notably, the majority of ischemic strokes occur within the first 2 days postprocedure, so it is likely that factors surrounding the procedure are the ones contributing significantly to the risk [18].

The reduction in disabling stroke observed in our meta‐analysis aligns with previous findings. Butala et al. demonstrated a 13% relative decrease in disabling stroke with embolic protection use, and in patients with a history of prior stroke, the risk was 40% lower [19]. However, our results also contradict Kolte et al., who found no significant reduction in mortality associated with the use of CEPDs, as well as a more recent propensity‐matched analysis conducted in 2024 [20, 21]. Other results, such as AKI, bleeding, and vascular complications, are affected by a variety of factors and individual circumstances as well. These effects could be influenced by systemic embolism or inflammatory response mitigation, but may also be due to differences in patient selection or specific differences between procedures. Major vascular complications, TIA, PPM placement, and delirium were comparable between groups, indicating that CEPD use does not bring about a substantial difference in these outcomes.

Subgroup analysis showed that there are some contradictions in our findings between RCTs and observational studies. We noticed that stroke, disabling stroke, and mortality were significantly reduced in observational studies compared to RCTs. There may be several reasons for this; observational studies are inherently prone to problems like selection bias, confounding by indication, and unmeasured variables that may influence outcomes. On the other hand, RCTs reduce these biases through randomization but often involve smaller sample sizes and include only specific patient groups, which may not reflect typical clinical settings. RCTs are also usually done in centers with more experienced operators and strict protocols, which might reduce the visible difference between patients with and without CEPDs. This suggests that CEPDs may work better in real‐world settings than in controlled trial environments.

The Sentinel device was used in the majority of the trials in this meta‐analysis. Although it is the most widely studied CEPD, there are several flaws in its design. It only protects the brachiocephalic and left common carotid arteries, leaving the left vertebral artery, and therefore the posterior circulation, unprotected. Since the left vertebral artery also generally has a greater vascular territory, and the volume and size of embolic debris passing through it during TAVR is comparable to that in the protected arteries, its exclusion from the Sentinel's protection is particularly worrisome [22, 23]. The Sentinel is available in only one filter size, which may not fit all arch anatomies optimally. The procedure also requires separate arterial access, which adds complexity and increases fluoroscopy time. These factors can contribute to a learning curve that may hinder widespread adoption and limit consistent benefit across different centers.

In addition to the Sentinel device, several other CEPDs have been developed to enhance stroke prevention during TAVR procedures. The TriGUARD 3 device is designed to provide full cerebral protection by covering all three aortic arch vessels, and its safety during TAVR was established in the REFLECT II trial [24]; however, it did not meet its pre‐specified primary efficacy endpoint, which suggests that further research is needed to establish its clinical benefit. The Emboliner Total Embolic Protection Catheter, EMBOL‐X device, and Emblok embolic protection systems are some other CEPDs that have a different mechanism from Sentinel. Instead of capturing debris, they deflect it [25]. Among these, the Emblok system has shown promising results, but large‐scale randomized trials are still underway to evaluate its true clinical benefit [26]. Time will tell whether these newer devices can overcome the limitations of earlier CEPDs and establish themselves in routine practice.

From a financial perspective, the Sentinel device adds approximately $2000 per procedure, which can increase overall healthcare expenditures, especially when extrapolated to national or global TAVR volumes. Wolfrum et al. argue that while routine use may not be cost‐effective across all patient groups, it might be justifiable in younger patients with longer life expectancies and greater potential to benefit from stroke prevention [7]. In one cost‐effectiveness model, the mean predicted quality‐adjusted life expectancy at 5 years was 29.4 months for patients receiving CEPD versus 28.7 months for those without [27]. The use of CEPDs also requires specific operator training to maximize safety and efficacy, and not all centers may have the resources or patient volume to maintain such proficiency.

Our study offers several strengths that enhance the reliability and applicability of its findings. By combining data from both RCTs and observational studies, we captured a broad spectrum of clinical practice and patient populations. This scale improves statistical power and allows for a more comprehensive assessment of clinical endpoints, and also shows us how CEPDs perform in both the controlled settings of clinical trials as well as real‐world settings.

However, this study has some limitations as well. Heterogeneity in study design, patient populations, and endpoint definitions may introduce variability in pooled estimates. The observational studies included are also susceptible to confounding and selection bias, even after performing statistical adjustments. Additionally, device‐related data were limited to the Sentinel system, preventing generalizability to other CEPDs currently in development or use. The absence of individual patient‐level data also restricted the ability to perform subgroup analyses to identify populations that might derive the most benefit. Lastly, publication bias cannot be entirely excluded, as studies with neutral or negative findings may be underrepresented in the literature.

## 5 Conclusion

CEPD use during TAVR appears to reduce the risk of stroke and other complications such as AKI and bleeding, particularly in observational studies. However, this benefit was not consistently observed in RCTs, including the recent negative PROTECTED TAVR trial. Given the high cost and lack of robust RCT evidence, routine use of CEPDs remains debatable and should be guided by future trials targeting high‐risk subgroups.

## Ethics Statement

No ethical approval was required for this study design, as all data were obtained from publicly available sources.

## Conflicts of Interest

The authors declare no conflicts of interest.

## Supporting information

Supple.

Re_Requested Supporting Figures and Tables – CCD70146.

## Data Availability

The data that support the findings of this study are available from the corresponding author upon reasonable request.
