# Supplementary material for: Efficacy of Cerebral Embolic Protection Device in Transcatheter Aortic Valve Replacement: A Systematic Review and Meta‐Analysis
Source: Catheter Cardiovasc Interv. 2025 Sep 5;106(5):2996–3007. doi: 10.1002/ccd.70146 (PMC12584568; doi:10.1002/ccd.70146)
Supplement: Supplementary file 1 — Supple. [file CCD-106-2996-s001.docx]

**Table 1: Search Strategy.**

| ***Database*** | ***Search String*** |
| --- | --- |
| ***PubMed*** | *("Transcatheter Aortic Valve Replacement"[Mesh] OR "TAVR" OR "TAVI" OR "transcatheter aortic valve implantation" OR "transcatheter aortic valve replacement")*  *AND*  *("Embolic Protection Devices"[Mesh] OR "cerebral embolic protection" OR "cerebral protection device" OR "Sentinel device" OR "embolic protection device" OR "CEPD")* |
| ***Embase*** | *('transcatheter aortic valve implantation'/exp OR 'transcatheter aortic valve replacement' OR TAVI OR TAVR)*  *AND*  *('cerebral embolic protection device'/exp OR 'cerebral protection device' OR 'embolic protection device' OR 'Sentinel device' OR CEPD)*  *AND*  *('randomized controlled trial'/exp OR 'randomised controlled trial' OR RCT)* |
| ***Scopus*** | *(TITLE-ABS-KEY("transcatheter aortic valve replacement" OR "transcatheter aortic valve implantation" OR TAVR OR TAVI))*  *AND*  *(TITLE-ABS-KEY("cerebral embolic protection" OR "cerebral protection device" OR "embolic protection device" OR "Sentinel device" OR CEPD))*  *AND*  *(TITLE-ABS-KEY("randomized controlled trial" OR "randomised controlled trial" OR RCT))* |


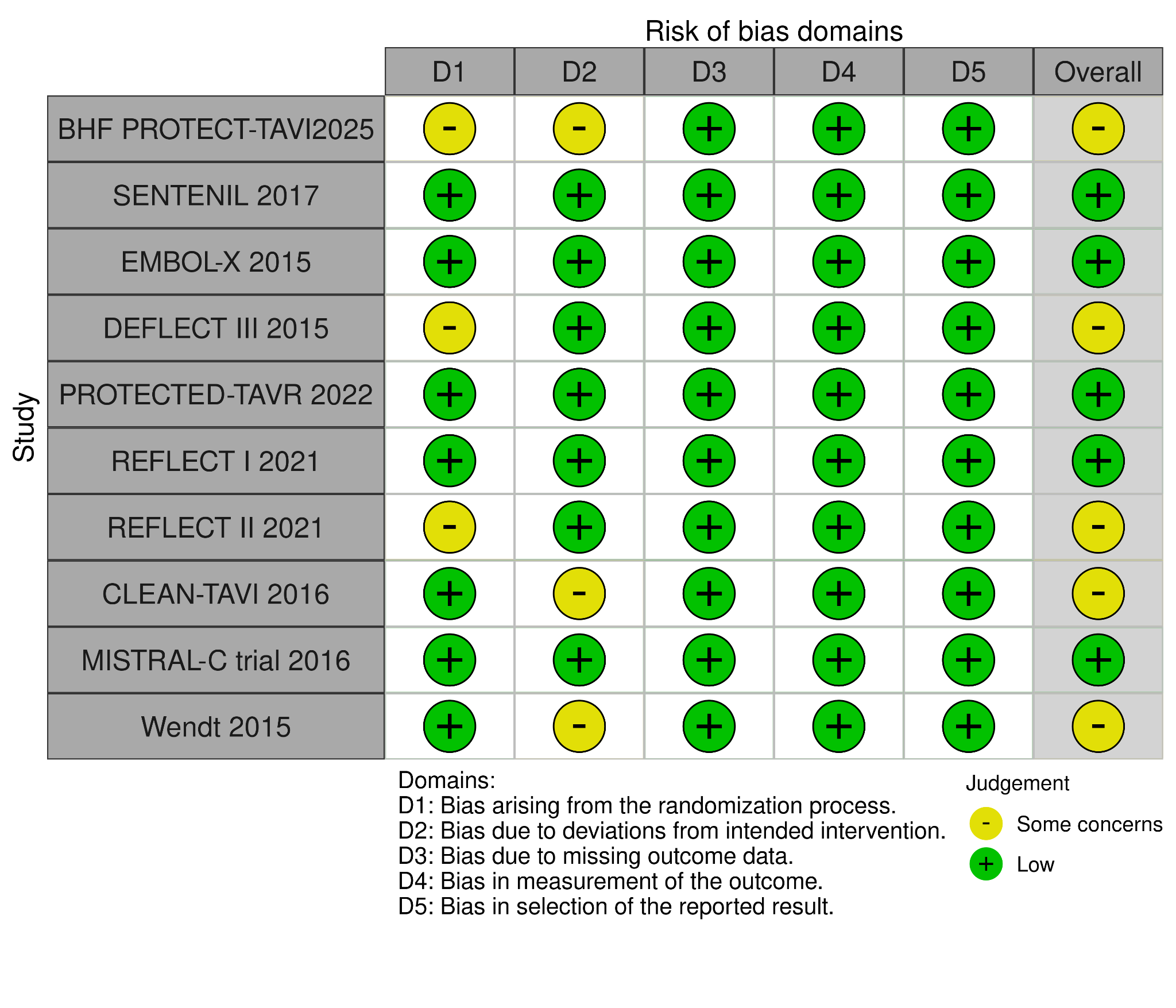
**Figure S1:** Traffic light plot showing risk of bias assessment for RCTs using the RoB 2.0 tool.


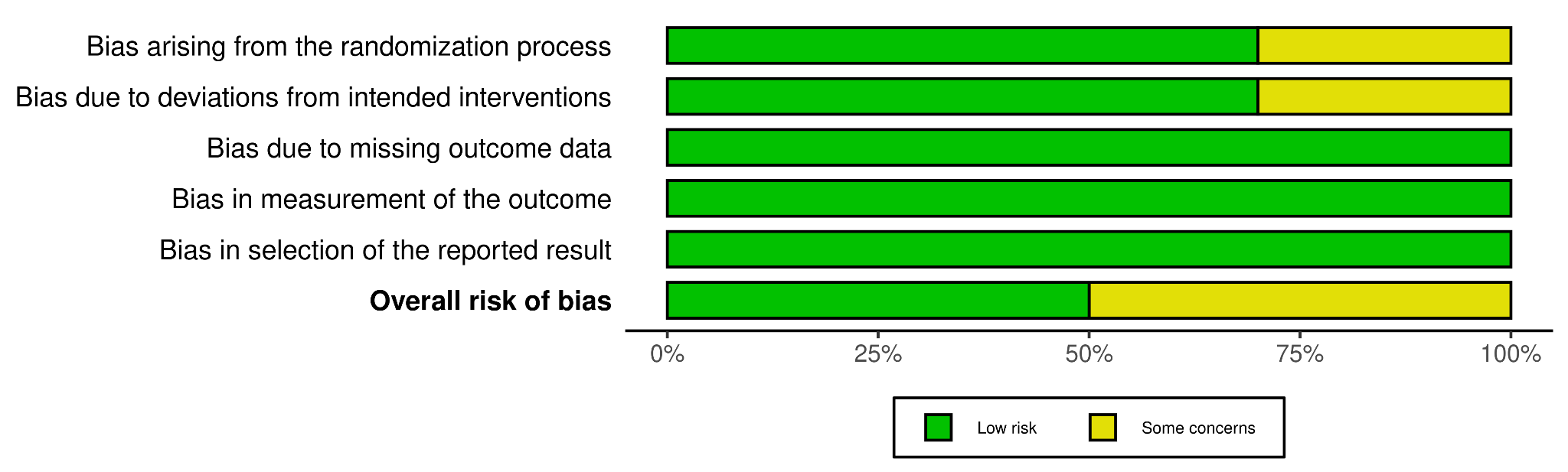
**Figure S2:** Summary light plot showing risk of bias assessment for RCTs using the RoB 2.0 tool.


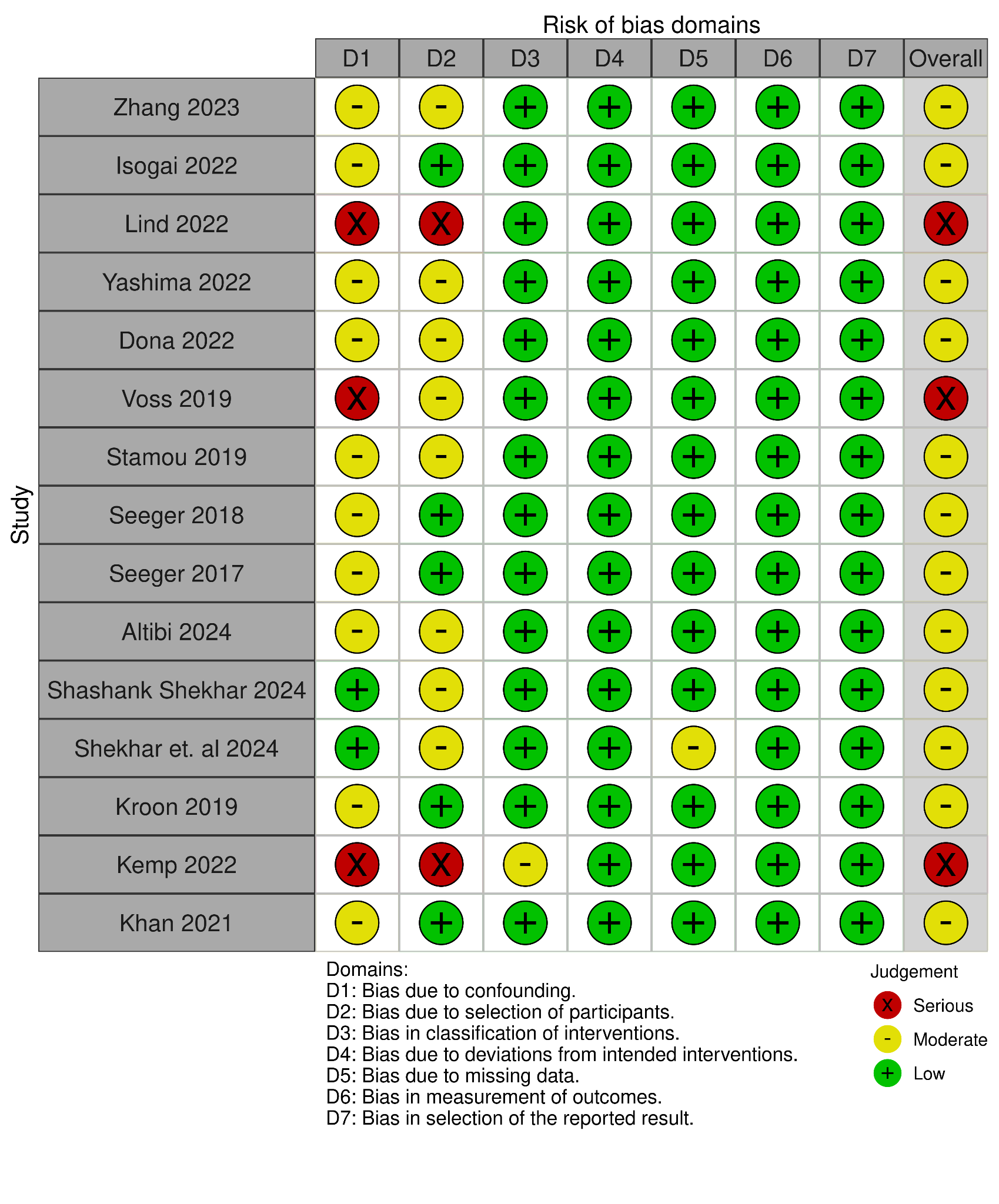
**Figure S3:** Traffic light plot showing risk of bias assessment for observational studies using the ROBINS I tool.


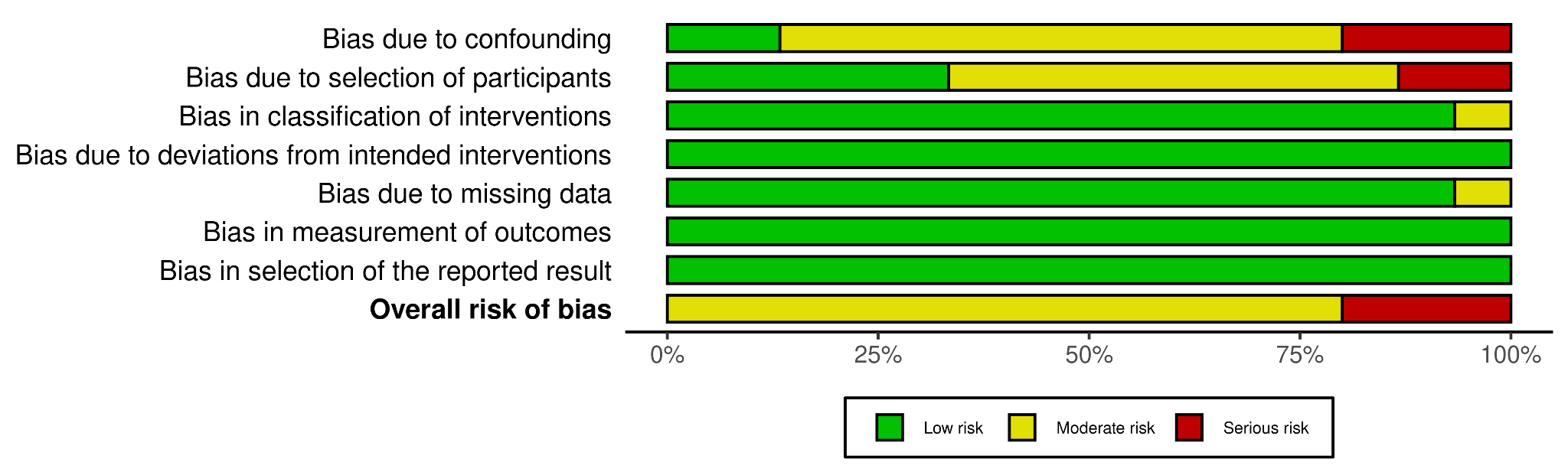


**Figure S4:** Summary light plot showing risk of bias assessment for observational studies using the ROBINS I tool.

**
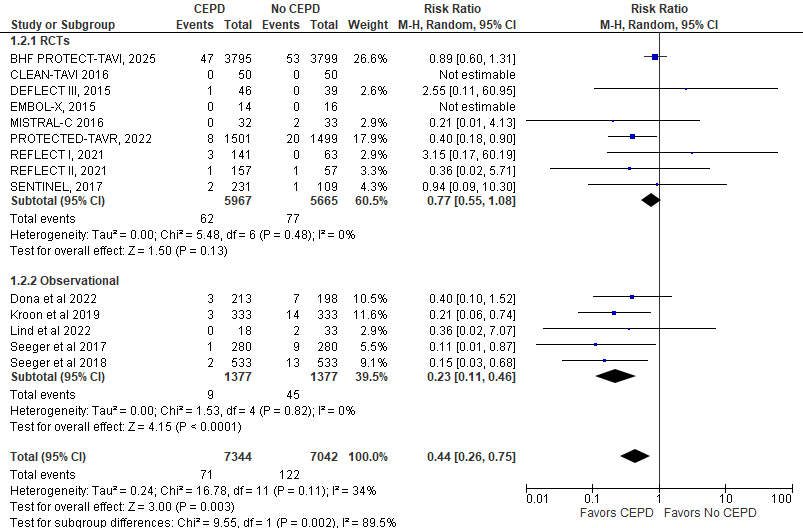
**

**Figure S5.** Forest plot comparing the outcome of **disabling stroke** for patients who underwent TAVR with cerebral embolic protection device (CEPD) to those who underwent TAVR without the use of CEPD.

**
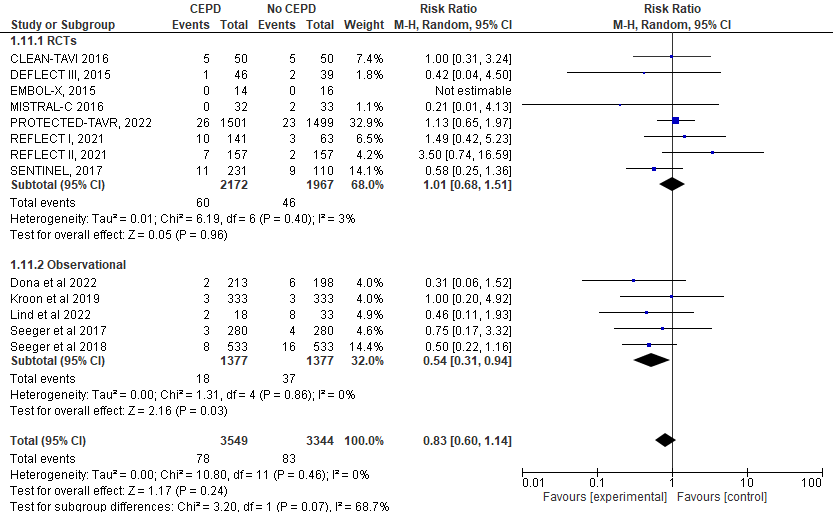
**

**Figure S6.** Forest plot comparing the outcome of **non-disabling stroke** for patients who underwent TAVR with cerebral embolic protection device (CEPD) to those who underwent TAVR without the use of CEPD.

**
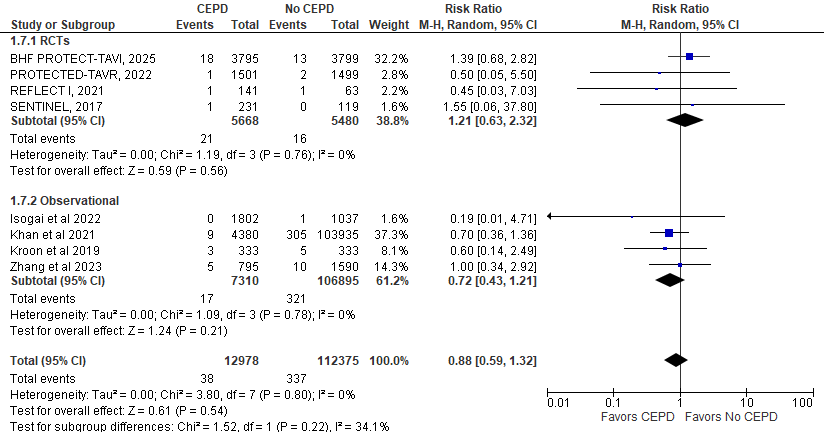
**

**Figure S7.** Forest plot comparing the outcome of **transient ischemic attack** for patients who underwent TAVR with cerebral embolic protection device (CEPD) to those who did not receive CEPD

**
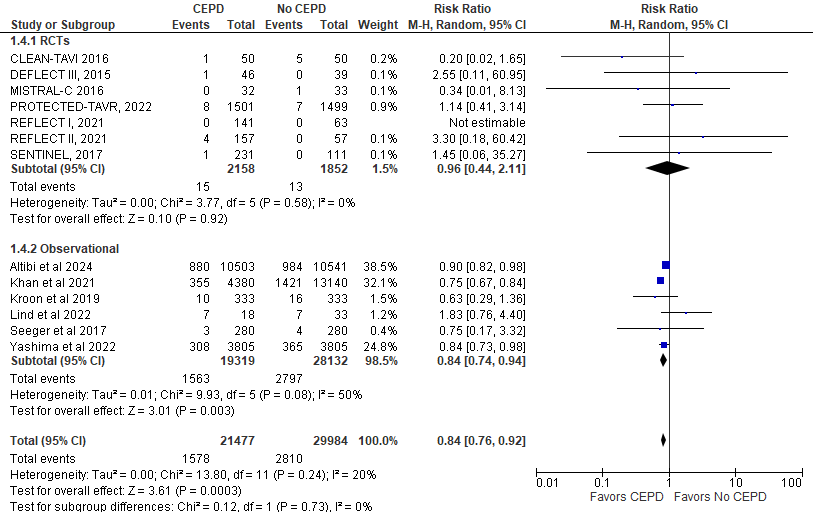
**

**Figure S8.** Forest plot comparing the outcome of **acute kidney injury** for patients who underwent TAVR with cerebral embolic protection device (CEPD) to those who underwent TAVR without the use of CEPD.

**
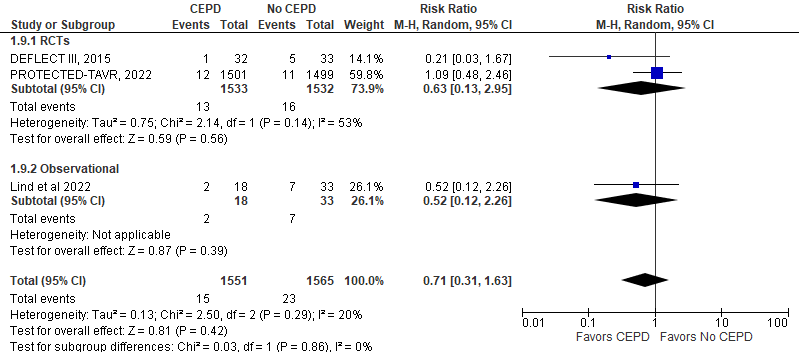
**

**Figure S9.** Forest plot comparing the outcome of **delirium** for patients who underwent TAVR with cerebral embolic protection device (CEPD) to those who underwent TAVR without the use of CEPD.

**
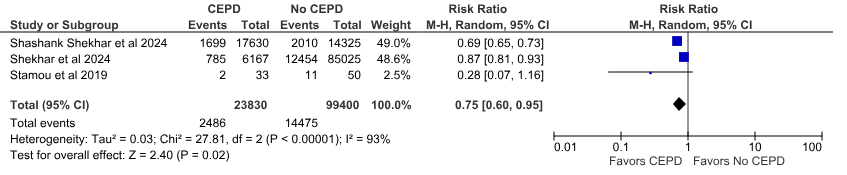
**

**Figure S10.** Forest plot comparing the outcome of 30-Days readmission for patients who underwent TAVR with cerebral embolic protection device (CEPD) to those who underwent TAVR without the use of CEPD.

**
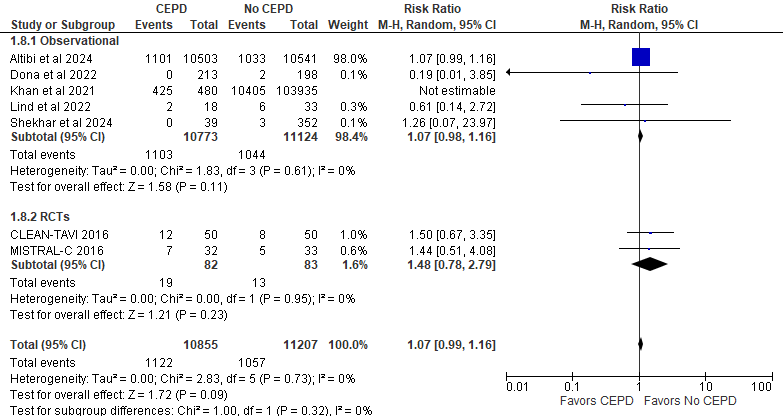
**

**Figure S11.** Forest plot comparing the outcome of **permanent pacemaker placement** for patients who underwent TAVR with cerebral embolic protection device (CEPD) to those who underwent TAVR without the use of CEPD.


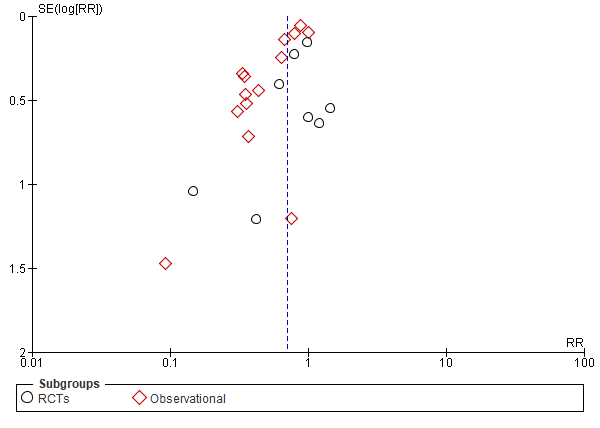


**Figure S12**. Funnel plot comparing the outcome of **stroke** for patients who underwent TAVR with cerebral embolic protection device (CEPD) to those who underwent TAVR without the use of CEPD.


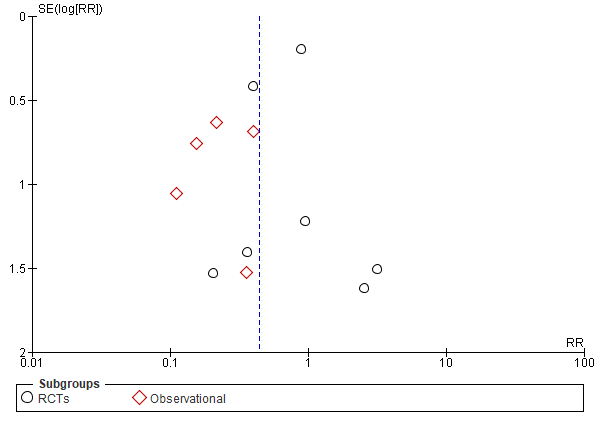


**Figure S13.** Funnel plot comparing the outcome of **disabling stroke** for patients who underwent TAVR with cerebral embolic protection device (CEPD) to those who underwent TAVR without the use of CEPD.


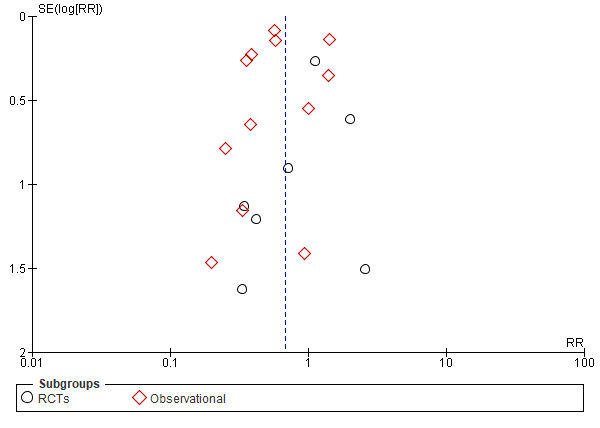


**Figure S14.** Funnel plot comparing the outcome of all cause **mortality** for patients who underwent TAVR with cerebral embolic protection device (CEPD) to those who underwent TAVR without the use of CEPD.


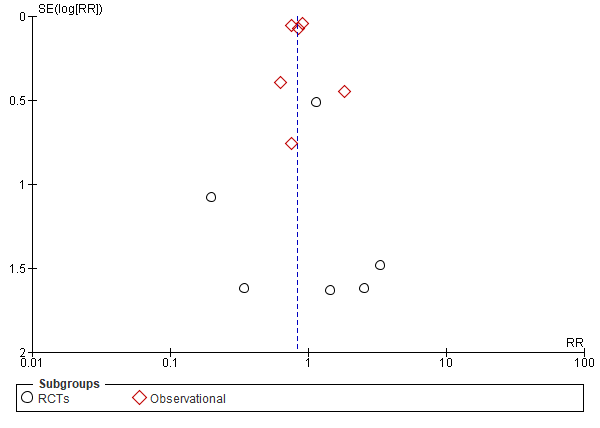


**Figure S15.** Funnel plot comparing the outcome of **acute kidney injury** for patients who underwent TAVR with cerebral embolic protection device (CEPD) to those who underwent TAVR without the use of CEPD.


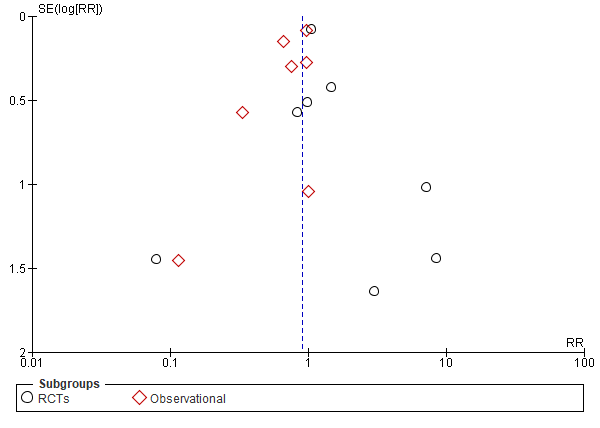


**Figure S16.** Funnel plot comparing the outcome of **major vascular complications** for patients who underwent TAVR with cerebral embolic protection device (CEPD) to those who underwent TAVR without the use of CEPD.


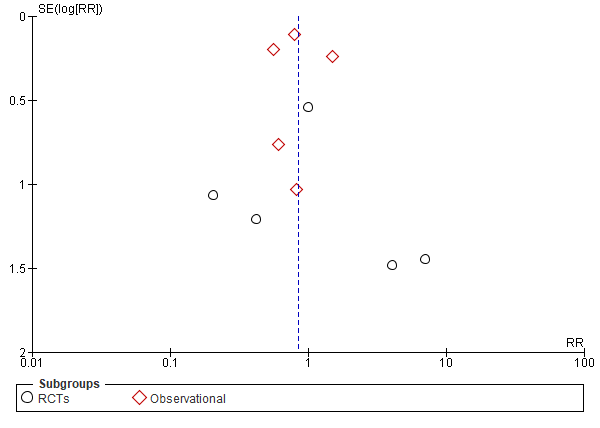


**Figure S17.** Funnel plot comparing the outcome of **major bleeding** for patients who underwent TAVR with a cerebral embolic protection device (CEPD) to those who underwent TAVR without the use of CEPD.


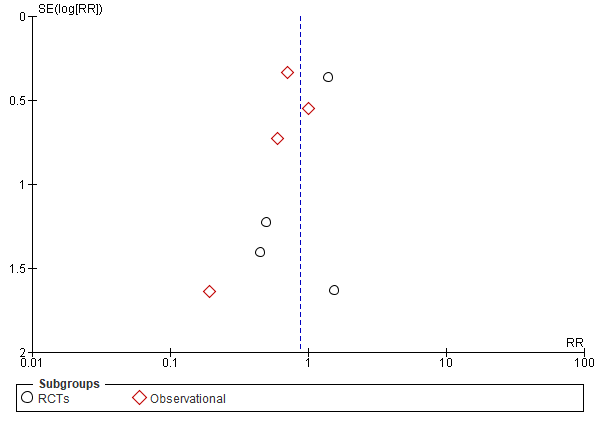


**Figure S18.** Funnel plot comparing the outcome of **transient ischemic attack** for patients who underwent TAVR with cerebral embolic protection device (CEPD) to those who did not receive CEPD


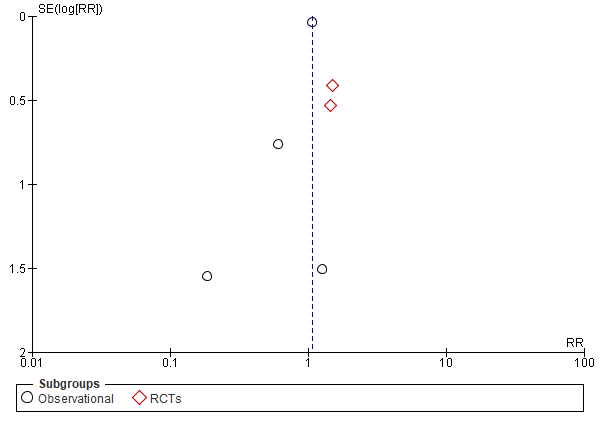


**Figure S19.** Funnel plot comparing the outcome of **permanent pacemaker placement** for patients who underwent TAVR with cerebral embolic protection device (CEPD) to those who underwent TAVR without the use of CEPD.


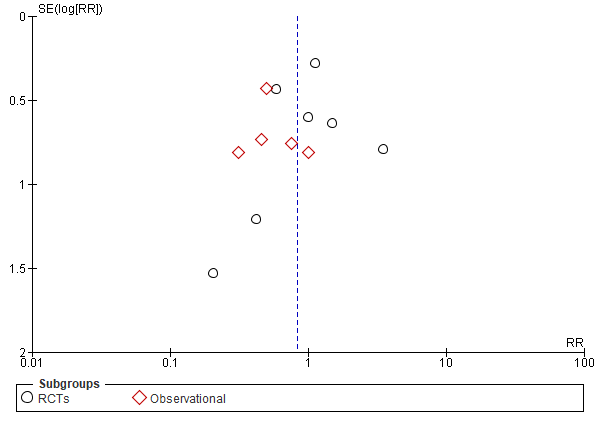


**Figure S20.** Funnel plot comparing the outcome of **non-disabling stroke** for patients who underwent TAVR with cerebral embolic protection device (CEPD) to those who underwent TAVR without the use of CEPD.


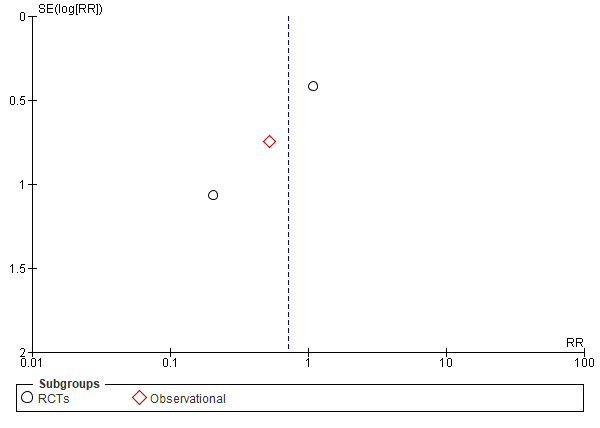


**Figure S21.** Funnel plot comparing the outcome of **delirium** for patients who underwent TAVR with cerebral embolic protection device (CEPD) to those who underwent TAVR without the use of CEPD.


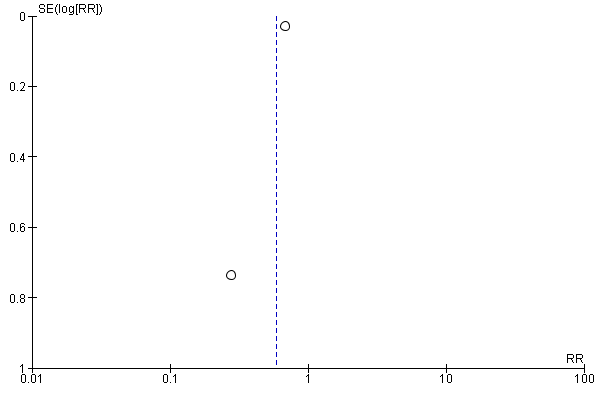


**Figure S22**. Funnel plot comparing the outcome of 30-Days readmission for patients who underwent TAVR with cerebral embolic protection device (CEPD) to those who underwent TAVR without the use of CEPD.
